# Adherence to Mediterranean Diet Is Associated With Better Glycemic Control in Children With Type 1 Diabetes: A Cross-Sectional Study

**DOI:** 10.3389/fnut.2022.813989

**Published:** 2022-03-04

**Authors:** Jesus Dominguez-Riscart, Nuria Buero-Fernandez, Ana Garcia-Zarzuela, Celia Morales-Perez, Ana Garcia-Ojanguren, Alfonso M. Lechuga-Sancho

**Affiliations:** ^1^Unidad de Endocrinología Pediátrica, Servicio de Pediatría, Hospital Universitario Puerta del Mar, Cádiz, Spain; ^2^Grupo de Inflamación, Nutrición, Metabolismo, y Estrés Oxidativo (INMOX), Instituto de Investigación e Innovación Biomédica de Cádiz (INiBICA), Cádiz, Spain; ^3^Servicio de Pediatría, Hospital Universitario Puerto Real, Puerto Real, Spain; ^4^Departmento Materno Infantil y Radiología, Facultad de Medicina, Universidad de Cádiz, Cádiz, Spain

**Keywords:** childhood, diabetes, glycemic control, Mediterranean diet (MD), nutrition, time in range, type 1 diabetes (T1D)

## Abstract

Type 1 diabetes (T1D) is a chronic condition, with increased morbidity and mortality, due to a higher rate of cardiovascular disease among other factors. Cardiovascular risk increases with the worse glycemic profile. Nutrition has a deep impact on diabetes control. Adherence to the Mediterranean diet (MD) has been shown to decrease cardiovascular risk in children and adults with obesity and adults with type 2 diabetes, but its impact on T1D children has been scarcely analyzed. We hypothesized that the degree of adherence to MD could relate to the increased time in range in children with T1D.

**Patients and Methods:**

Cross-sectional analysis involving two university hospitals. We measured the adherence to MD with the Mediterranean Diet Quality Index for children and teenagers (KIDMED) questionnaire, which is a validated tool for this purpose. A score of <5 indicates poor adherence to MD, while a good adherence is indicated by a score of >7. Demographic and clinical data were registered on the same day that the questionnaire was taken, with informed consent. Additionally, the patients' ambulatory glucose profiles (AGPs), were registered from the participants' glucose monitors (continuous or flash devices), and daily insulin needs were recorded from patients' insulin pumps (n=28). Other cardiovascular risk factors such as lipid profile, vitamin D levels, and other biochemical parameters were registered from a blood test, performed 2 weeks before recruitment, as part of the patients' annual screening.

**Results:**

Ninety-seven patients (44 girls), with an average age of 11.4 years (± 3.01), were included. Seventy-one of them were on multiple daily injection regimens, and all had either continuous or flash glucose monitoring. Fifty-three had HbA1c levels of <7.5%, while only 21 had a time in range (TIR) of >70%. Contingency analysis showed that the odds of having HbA1c <7.5% increase in children with KIDMED score of >7 (O.R. 2.38; ICR 1.05–5.41; *p* = 0.036). Moreover, the KIDMED score and the HbA1c levels were negatively correlated (R: −0.245; *p*-value: 0.001), while the KIDMED score and TIR showed a positive correlation (R: 0.200; *p*-value: 0.009).

**Conclusions:**

Our data suggest that adherence to MD may contribute to better glycemic control in children. This should be taken into account at the time of nutritional education on T1D patients and their families.

## Introduction

Type 1 diabetes (T1D) is a chronic autoimmune disease characterized by the absence of insulin production due to immune-mediated destruction of the pancreatic beta-cells. Patients with type 1 diabetes have an increased cardiovascular risk, potentiated by poor glycemic control (1, 2). The definition of optimal glycemic control and the recommended targets are frequently being updated as new parameters/indicators emerge. This is due to growth in the use of diabetes technologies such as Continuous Glucose Monitors, Insulin Pumps, and integrated systems (3). The latest recommendations from the American diabetes association (ADA) set an HbA1C goal of <7% (53 mmol/mol) across all the pediatric ages, although this must be individualized (4), while the International Consensus on Time in Range additionally recommends a time in Range (TIR) of at least 70% (5). This second goal is relevant since HbA1c does not reflect glycemic excursions leading to acute events, which have been linked to both micro- and macrovascular diabetic complications (6).

To achieve these glycemic control goals, patients, and their family/caregivers are instructed in specific diabetic educational programs in the management of the four cornerstones of their therapy: insulin needs and administration, interpreting glucometer/sensors' information, considerations regarding both, physical exercise, and nutrition. Nutritional education is based on healthy eating habits. Besides tailoring to individual tastes and needs, special attention is paid to train on carbohydrate-counting, which is essential for prandial insulin bolus calculation (7, 8).

The Mediterranean diet (MD) pattern has proven to have benefits on both cardiovascular risk and diabetes control. On the one hand, it has been shown to decrease mortality in patients with cardiovascular risk (9), and the risk of stroke in the general population (10). On the other, it is in line with the standards of medical care that is established for T1D (11), since it is based on frequent consumption of fresh fruits and vegetables, legumes, olive oil, whole grains, nuts, and fish, which are estimated to provide considerable amounts of antioxidants, carotenoids, polyunsaturated lipids, and fiber (12–14), while the consumption of meat and processed foods are marginal. The MD provides numerous healthy dietary components promoting health and well-being. It also reduces the likelihood of chronic conditions such as heart disease, type-2 diabetes (T2D), or cancer in obese populations (15).

The KIDMED score is useful and is a widely accepted tool to assess adherence to MD in children and adolescents (16), which has recently been updated for the Spanish population (17). It is a simple consumption-frequency questionnaire on the MD indicators. It has been used in studies showing that higher adherence to MD is associated with a reduced incidence of obesity and prevents cardiovascular risk in children and adolescents (18). Nevertheless, the effects of MD on children and adolescents with T1D have been scarcely studied, and to the best of our knowledge, only once has its association with glycemic control been assessed (19, 20). Thus, we aimed to monitor the adherence to MD in children and adolescents with T1D and to test whether it relates to glucose control, particularly with TIR, which has not previously been analyzed.

## Patients and Methods

### Study Design and Participants

We performed a two-center cross-sectional study targeting the whole sample of children and adolescents with T1D, who regularly followed up at the diabetes units of both centers. These centers are located in southern Spain, a region included in the Mediterranean lifestyle. All participants were on carbohydrate counting. Recruitment and data acquisition were performed from January 2020 to January 2021. Every patient with ages between 4 and 16 years, diagnosed with T1D, using either continuous glucose monitoring or intermittent glucose monitoring devices, and a disease duration of more than 1 year were included. Patients requiring additional specific nutritional therapy, such as those with microalbuminuria, hyperlipidemia, chronic renal failure, or celiac disease were excluded.

### Data Acquisition

Following the latest international society for pediatric and adolescent diabetes (ISPAD) recommendations, we annually screened every patient with T1D over 11 years of age for micro and macrovascular complications, as well as other cardiovascular risk factors (21). According to our Unit's protocol, we expanded these recommendations to every patient with T1D at diagnosis and yearly, thereafter. Anthropometrical data, along with other clinical and biochemical parameters (venous HbA1c, and lipid profile), were registered coinciding with this annual screen visit, by the pediatric endocrinologist in charge of the patient. Total daily insulin dose was registered only from patients with Continuous Subcutaneous Insulin Infusion (CSII) therapy.

At this same visit, every participant was informed about the study and was asked to participate. After informed consent was granted, we passed the KIDMED questionnaire to them, while downloading the patients' glucose monitors [Guardian Connect sensor (Medtronic, Northridge, CA, USA) or FreeStyle Libre 2 (Abbott Diabetes Care, Oxon, UK)]. The KIDMED score and the ambulatory glucose profile (AGP) parameters [mean blood glucose, standard deviation, deviation coefficient, % of the time in range (70–180 mg/dl), and % of time above and below range], of the last 3 months were recorded, for the study.

### Statistical Analysis

Descriptive analysis included mean and standard deviation for quantitative variables, while frequency and percentages for qualitative variables. We then divided the sample into two groups, according to their degree of adherence to MD (suboptimal adherence–KIDMED score <8–*vs*. optimal adherence–KIDMED score ≥ 8). The Kolmogorov-Smirnoff's test was used to study the distribution of each variable. Comparisons between both groups were performed by applying Student's *t*-test for unpaired samples when comparing quantitative variables since every variable followed a normal distribution. Qualitative variables were compared using chi-square. Contingency tables were created to calculate the odds ratio of meeting the goal of <7.5% of HbA1c, according to these two groups. We applied Pearson's correlation coefficient to explore the linear relationships between each explanatory variable and the KIDMED score. All statistical analyses were performed with the SPSS software, version 15.0 (SPSS Inc., Chicago, Illinois, USA). A significant difference was defined by a *p*-value under 0.05.

### Ethical Considerations

Written informed consent was obtained from every participant older than 12, and the parents/caregivers of those participants under 12. The study was performed following the Data Protection and Privacy Principles of the Spanish legislation and the European Union Regulations. The Institution's Ethics Committee supervised and approved the study protocol (#1305-N-20).

## Results

Ninety-seven patients were included, 53 (54, 6%) were male of ages ranging from 3 to 16.6 years. The mean age of the whole sample was 11.4 ± 8.04 years and 35 (36, 1%) were pre-puberal. Diabetes duration ranged from 1.1 years to 12.5, years with a mean of 5.9 ± 5 years. Seventy-one of them were on Multiple Daily Injections (MDI) therapy and 26 with Continuous Subcutaneous Insulin Infusion (CSII). The former group used an intermittent glucose monitor (Free Style Libre v.2), while the latter used the Medtronic's Enlite v3. Twelve of these were under Hybrid Close-Loop artificial pancreas. Clinical and biochemical characteristics of the whole sample and the comparison between optimal adherence to MD, and the suboptimal adherence to MD groups are summarized in Table 1.

**Table 1:** Descriptive analysis of the characteristics of participants according to MD adherence.

	**All patients**	Optimal adherence to MD^*^	**Suboptimal adherence to MD**	***P*-value**
*N*	97	51	46	-
Clinical parameter				
Sex (Male) [*n* (%)]	53 (54.6%)	27 (52.8%)	26 (56.5%)	0.82
Age (years)	11.4 ± 8.04	11.2 ± 8.3	11.7 ± 10.1	0.61
BMI (Kg/m^2^)	20.2 ± 3.7	20.16 ± 3.5	20.4 ± 4.4	0.85
BMI (z-score)	0.45 ± 1.03	0.33 ± 0.97	0.57 ± 1.08	0.28
Obesity [*n* (%)]	6 (6.1%)	2 (4%)	4 (8.6%)	0.41
SBP (mmHg)	107.4 ± 0.17	107 ± 0.1	107.6 ± 0.29	0.248
DBP (mmHg)	68.2 ± 0.58	69 ± 0.53	67.5 ± 0.65	0.374
Tanner stage [*n* (%)]				
I	35 (36.1%)	19 (37%)	16 (34.8%)	0.10
II	9 (9.3%)	7 (13.7%)	2 (4.3%)	
III	12 (12.4%)	8 (15.7%)	4 (8.7%)	
IV	9 (9.3%)	6 (11.8%)	3 (6.5%)	
V	32 (33%)	11 (21.6%)	21 (45.6%)	
Diabetes duration (years)	5.9 ± 5	5.1 ± 3.3	6.8 ± 6	0.16
Insulin therapy [*n* (%)]				
MDI [*n* (%)]	71 (73.1%)	38 (72.5%)	34 (74%)	0.59
CSII [*n* (%)]	26 (26.9%)	14 (27.5%)	12 (26%)	0.612
Total daily Insulin (UI/kg/day)	0.704 ± 0.54	0.69 ± 0.48	0.72 ± 0.50	0.372
AGP parameters				
TIR (%)	56.4 ± 16.3	60.4 ± 15.7	52.1 ± 16.02	0.023
TIR >70% [*n* (%)]	17 (21.3%)	11 (26.2%)	6 (15.8%)	0.11
TAR (%)	39.3 ± 16.7	36.8 ± 15.8	42.8 ± 17.4	0.11
TBR (%)	3.3 ± 3	2.7 ± 3	4 ± 4.3	0.127
VC (%)	35.6 ± 6.4	36.5 ± 5.7	34.6 ± 7.1	0.24
MBG (mg/dl)	170 ± 25.2	157 ± 20.3	174 ± 29.5	0.10
HbA1c (mmol/L)	9.21 ± 1.62	8.89 ± 1.10	9.68 ± 2.04	0.005
HbA1C (%)	7.4 ± 0.81	7.2 ± 0.55	7.7 ± 0.97	0.005
HbA1c <7.5% [*n* (%)]	53 (54.6%)	33 (64.7%)	20 (43.5%)	0.029
Total cholesterol (mg/dl)	169 ± 23	167.5 ± 23.91	172 ± 22.2	0.29
LDL-c (mg/dl)	96.8 ± 19.1	94.7 ± 19.4	98.7 ± 11.8	0.24
HDL-c (mg/dl)	60 ± 12.4	60.6 ± 11.86	59.5 ± 13.1	0.64
Triglycerides (mg/dl)	65 ± 19.9	63.02 ± 15.7	68.8 ± 23.08	0.35
KIDMED score	7.04 ± 2.03	9 ± 1.09	5.6 ± 1.19	<0.001
KIDMED-score <5 [*n* (%)]	7 (7.2%)	0 (0%)	7 (7.2%)	<0.001
KIDMED score 5–7 [*n* (%)]	39 (40.2%)	0 (0%)	39 (40.2%)	
KIDMED score ≥ 8 [*n* (%)]	51 (52.6%)	51 (52.6%)	0 (0%)	

* *Optimal KIDMED score was defined by a value ≥ 8. Values are expressed as Mean ± Standard deviation, unless otherwise stated. BMI, body mass-index; SBP, Systolic blood pressure; DBP, Diastolic blood pressure; Hb1Ac, glycated hemoglobin; T1D, Type 1 diabetes; TAR, Time above range; TIR, Time in range; MDI, Multiple Daily Injections; CSII, continuous subcutaneous insulin infusion; AGP, Ambulatory Glucose Profile; VC, variation coefficient; MBG, mean blood glucose; MD, Mediterranean diet*.

The mean KIDMED score value of the sample was 7.04 ± 2.03; the optimal adherence group had a mean score of 9 ± 1.09 *vs*. 5.6 ± 1.2 for the suboptimal adherence group (*p* < 0.01). Only seven of the 46 children in the suboptimal adherence group had a score <5, which is the definition of poor adherence.

**Figure 1:**
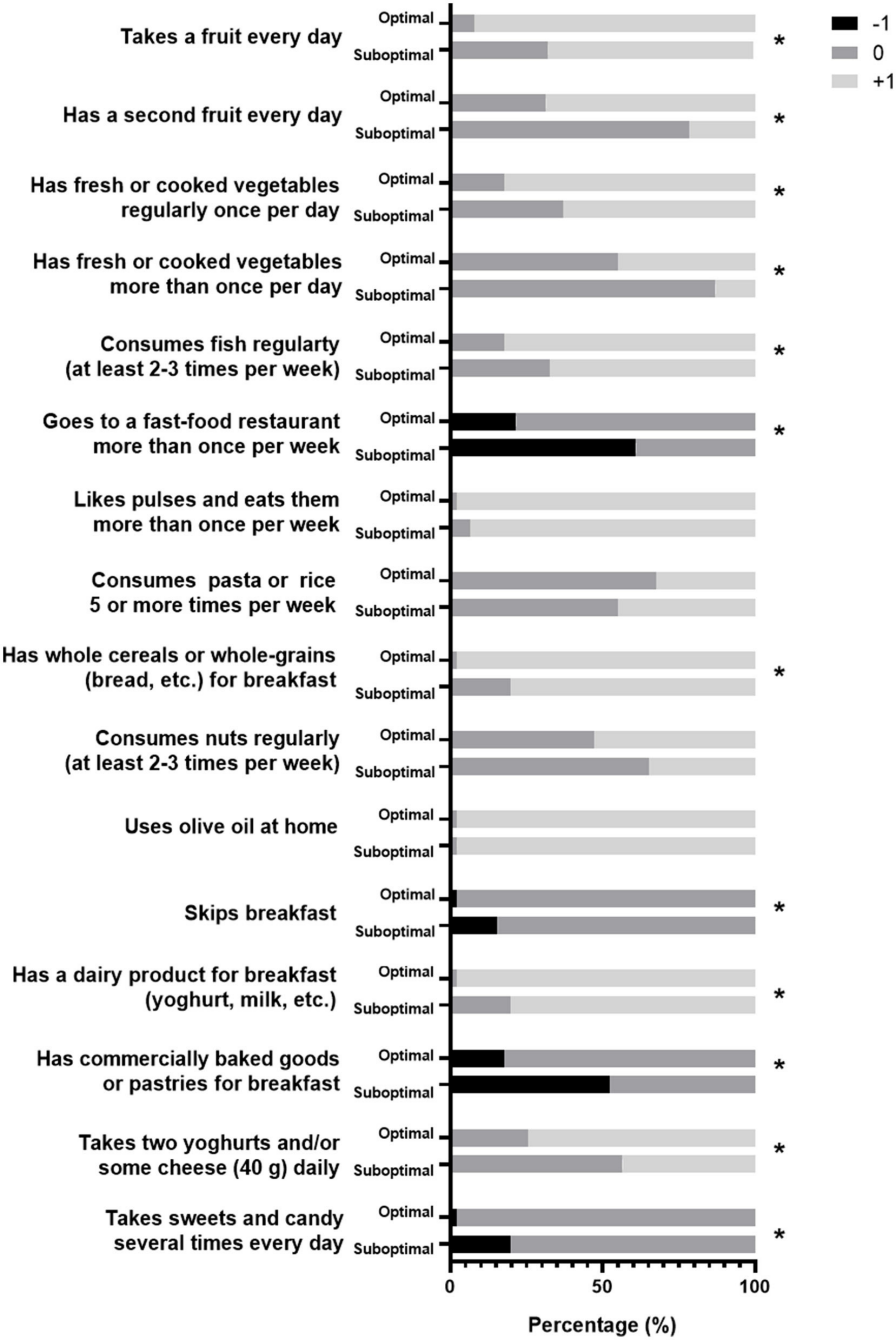
The graph showing how the different groups answered the KIDMED questionnaire. Black bars show answers that are subtracted from the score, in white are the ones that are added, and in gray are the ones that do not make a difference. * Show different answers (*p* <0.05) between groups.

The group with optimal adherence to the MD recommendations scored more points that are associated with eating fruit and vegetable, as well as more frequently consuming fish, whole cereals/grains, and dairy products. On the contrary, the suboptimal adherence to MD group lost points by going more frequently to fast-food restaurants or more frequently consuming commercially baked goods or pastries for breakfast, as well as more frequent skipping of breakfast, or eating sweets and candies more than once a day (Figure 1).

No differences were found between the groups in any of the anthropometrical variables, insulin therapy, or in the lipid profile variables that were analyzed. Regarding glucose control parameters, the mean HbA1c percentage of the whole sample was 7.4 ± 0.81, and the number of children complying with ADA's criteria for optimal control (HbA1c <7.5%) was 53 (54.6%), 33 of them were in the optimal adherence to MD group, while 20 in the suboptimal adherence (*p* = 0.029). In contrast, only 17 patients (21%) matched the ISPAD's additional criteria for optimal control (TIR > 70%); 11 in the optimal adherence group *vs*. six in the suboptimal adherence group (*p* = 0.11). However, TIR was higher in the group with a KIDMED score of ≥ 8 (60.4 ± 15.70 *vs*. 52.1 ± 16.02%; *p* = 0.023). The venous HbA1c was lower in the optimal adherence to MD group (7.2 ± 0.55 *vs*. 7.7 ± 0.97; *p* = 0.05). No other differences between groups were found in any of the remaining AGP values. The odds ratio of having HbA1c of <7.5% increase in children with KIDMED score of ≥ 8 (O.R. 2.38; ICR 1.05–5.41; *p* = 0.036).

About the correlation analyses for KIDMED score, we found that the adherence to the MD, positively correlated with TIR (R = 0.305; *p* = 0.006), and negatively correlated to Hb1Ac (R = −0.345; *p* < 0.001), mean blood glucose (R = −0.252; *p* = 0.022), and LDL-c (R = −0.209; *p* = 0.04). Data is shown in Table 2.

**Table 2:** Correlation between continuous variables and KIDMED score.

**Variable**	**Correlation Coefficient**	***P*-value**
Age (years)	−0.031	0.762
BMI (z-score)	−0.179	0.21
Diabetes duration (years)	−0.181	0.14
TIR (%)	0.305	0.006^*^
TAR (%)	−0.219	0.059
TBR (%)	−0.174	0.124
VC (%)	0.084	0.97
MBG (mg/dl)	−0.252	0.022^*^
HbA1C (%)	−0.345	<0.001^*^
Total cholesterol (mg/dl)	−0.166	0.103
LDL-c (mg/dl)	−0.209	0.04^*^
HDL-c (mg/dl)	−0.039	0.705
Triglycerides (mg/dl)	0.155	0.130

* *stands for a p value ≤ 0.05*.

## Discussion

Nutrition has a deep impact on diabetes control. Every patient with T1D, even those with the more advanced sensor-augmented pumps and hybrid close-loop pumps, still need to perform carbohydrate counting to obtain a prandial insulin recommendation from their systems. Indeed, a postprandial glycemia explains as much as 70% of daytime hyperglycemia in patients with T1D and/or with T2D (22, 23). Particularly, in patients with T1D, the postprandial glucose is highly predictive of HbA1c levels (24). It is of the utmost importance to control postprandial excursions since these are considered to be primarily responsible for the increased mortality due to cardiovascular causes, as well as increased retinopathy and nephropathy in patients with diabetes (25, 26). Thus, to achieve near-normal blood glucose levels, control of postprandial glucose is essential (27), particularly among those patients with better glycemic control (28).

Nutritional education for patients with T1D has largely focused on carbohydrate counting (29–31). However, there is an increasing awareness that different nutritional determinants are influencing postprandial blood glucose other than the amount of carbohydrates, such as the amount of protein and mono-unsaturated fatty acids and of total cholesterol (32). Additionally, different food-based approaches lead to improved food choices in people with diabetes, improving other cardiovascular risk factors, although the benefit of glucose control is still controversial (33).

The American Diabetes Association positions favorably toward the Mediterranean Diet as a pattern to be followed by the adults with diabetes (29), and children and adolescents with T1D who undergo structural nutritional training to the MD, to achieve a more favorable lipid profile after 6 months (19). Despite this evidence and these recommendations, the rate of children and adolescents with optimal adherence to the MD pattern is as low as 10% (34). Moreover, it has been found that as little as 2.8% of European children and adolescents had a high KIDMED score, while as many as 51.5% had a low score (20). In contrast, we found, in our population, a much higher adherence to the MD as measured by the KIDMED score, since 51 of the 97 children included scored higher than 7, and the mean score of our sample was 7.04. On the other hand, the rate of children scoring poor adherence (i.e., KIDMED of <5), was very low (7.2% of participants). This may be because we are located in a coastal location of Southern Spain, where olive oil, fresh fish, fruits, and vegetables are readily available, and the consumption of legumes is culturally high, and where the rate of adherence to the MD is generally high. Indeed, our MD adherence (Mean KIDMED score = 7.04) is very similar to what was described in the children from Southern Spain school in the epidemiological study by Arriscado et al., who found a Mean KIDMED score of 7.40 (35).

Zhong et al., previously found that patients with a higher KIDMED score had a lower HbA1c, lower total cholesterol and LDL-cholesterol, and higher HDL-cholesterol (20). Our data also show an improved HbA1c in the group with optimal adherence to MD. However, we found no differences between the groups in any of the lipid profile variables. This may be explained by the fact that our sample, in general, scored much higher in KIDMED than the study population of the referred study, hence, differences in lipid profile are more difficult to find. However, we did find a weak but significant negative correlation between the KIDMED score and LDL-cholesterol levels. This is in line with the results of a recent meta-analysis of the MD's effect on the primary prevention of cardiovascular disease. The study comprises 22 randomized controlled trials on primary prevention. The authors performed two different comparisons. In the first comparison, including nine clinical trials, they found with low or very low-quality evidence, little or no effect on LDL or HDL cholesterol or triglycerides. Similarly, in the second comparison (13 clinical trials), they show with moderate-quality evidence, a possible small reduction in LDL cholesterol (−0.15 mmol/L, 95% CI −0.27 to −0.2) (10).

As the technology for continuous glucose monitoring has evolved, improved, and spread, different glucose control metrics have arisen, which reflect glucose excursions better than HbA1c. The TIR, considered as the percentage of time that a patient spends with glucose levels between 70 and 180 mg/dl (3.9–10 mmol/L), provides more actionable information than HbA1c alone, and it has been internationally agreed that the primary goal for effective and safe glucose control should be to increase the patients' TIR (6). To the best of our knowledge, the MD's effect on TIR has not yet been analyzed. Our results show that higher adherence to MD correlates with a higher TIR in children with T1D and adolescents without correlation with increased TBR, supporting the benefit of the MD on glycemic control.

The main differences between groups were that the ones with optimal adherence to MD more frequently ate fresh fruits, vegetables and fish, and whole cereals/grains and dairy products, while the group with suboptimal and poor adherence to MD, more frequently ate at fast-food restaurants, and sweets/candies, and more frequently skipped breakfast or consumed commercially baked products or pastries as part of this meal. Of note, these are differences that may well be addressed as part of a diabetes structured educational program in nutrition, besides carbohydrate counting. Moreover, recommendations along these lines may also apply to populations with eating habits and lifestyles that are far from MD, as well as those located in latitudes far from the Mediterranean.

Our study is observational, thus, unable to attribute causation. To avoid selection bias, we included all of our cohort of patients complying with the inclusion criteria, and we found no differences between groups in any of the anthropometrical parameters. Moreover, we have adopted internationally accepted cut-off levels for sorting between optimal and suboptimal adherence to MD, as well as the main outcome values classifying glucose control (HbA1c and TIR). To avoid information bias, data acquisition was uniform for every patient included. The questionnaires were answered by participants before they were informed of the results of the information on their glucose sensors, and data on glucose control parameters, as well as the total daily insulin that were directly extracted from their systems. Since we found no differences between groups in lipid profile variables, such as BMI or blood pressure, it seems unlikely to have incurred confounding bias. To provide the study with some external validity, we have included patients from two different centers. However, both are located closely in the same province of Southern Spain, and this may limit the generalization of our results, even if the sample size is comparable to similar studies.

In summary, our data suggest that children with T1D have lower HbA1c and higher TIR when their nutritional pattern adheres to the Mediterranean diet, while the correlation with lower LDL-cholesterol levels was mild. Increasing adherence to MD may be achieved by increasing the frequency of consuming fruit, vegetables, fish, whole grains and cereals, and dairy products. Further recommendations should be to avoid skipping breakfast and to avoid pastries and commercially baked products for breakfast. It remains to be determined if a nutritional intervention in this way will effectively improve glucose control in those patients with T1D and poor adherence to MD. Our data advocate for such an intervention in a prospective study.

## Data Availability Statement

The raw data supporting the conclusions of this article will be made available by the authors, without undue reservation.

## Ethics Statement

As a study involving human participants, it was reviewed and approved by Comité de Ética de la Investigación de Cádiz. Written informed consent to participate in this study was provided by the participants' legal guardian/next of kin.

## Author Contributions

JD-R and AL-S conceptualized and designed the study, contributed to the clinical recruitment of patients, supervised the database, performed statistical analyses, drafted the initial manuscript, revised it, and wrote the final version. NB-F and CM-P contributed to the clinical recruitment of patients and reviewed and revised the manuscript. AG-Z and AG-O acquired data and critically revised the manuscript for important intellectual content. AL-S coordinated and supervised the procedure and data collection and wrote the final version of the manuscript. All the authors approved the final manuscript as submitted and agree to be accountable for all aspects of the work.

## Conflict of Interest

The authors declare that the research was conducted in the absence of any commercial or financial relationships that could be construed as a potential conflict of interest.

## Publisher's Note

All claims expressed in this article are solely those of the authors and do not necessarily represent those of their affiliated organizations, or those of the publisher, the editors and the reviewers. Any product that may be evaluated in this article, or claim that may be made by its manufacturer, is not guaranteed or endorsed by the publisher.
